# Hematopoietic Cell Kinase (HCK) Is Essential for NLRP3 Inflammasome Activation and Lipopolysaccharide-Induced Inflammatory Response *In Vivo*

**DOI:** 10.3389/fphar.2020.581011

**Published:** 2020-09-15

**Authors:** Xiangxi Kong, Yajin Liao, Lujun Zhou, Ying Zhang, Jinbo Cheng, Zengqiang Yuan, Shukun Wang

**Affiliations:** ^1^ The Brain Science Center, Beijing Institute of Basic Medical Sciences, Beijing, China; ^2^ Center on Translational Neuroscience, College of Life & Environmental Science, Minzu University of China, Beijing, China; ^3^ Laboratory of Oral Microbiota and Systemic Diseases, Shanghai Ninth People’s Hospital, Shanghai Jiao Tong University School of Medicine, Shanghai, China

**Keywords:** nod-like receptor family protein 3, inflammasome, hematopoietic cell kinase, macrophage, microglia, A419259

## Abstract

Activation of the NLRP3 inflammasome results in caspase 1 cleavage, which subsequently leads to IL-1*β* and IL-18 secretion, as well as pyroptosis, and aberrant activation of the inflammasome is involved in several diseases such as type 2 diabetes, atherosclerosis, multiple sclerosis, Parkinson’s disease, and Alzheimer’s disease. NLRP3 activity is regulated by various kinases. Genetic and pharmacological inhibition of the hematopoietic cell kinase (HCK), a member of the Src family of non-receptor tyrosine kinases (NRTKs) primarily expressed in myeloid cells, has previously been shown to ameliorate inflammation, indicating that it may be involved in the regulation of microglia function. However, the underlying mechanism is not known. Hence, in this study, we aimed to investigate the role of HCK in NLRP3 inflammasome activation. We demonstrated that HCK silencing inhibited NLRP3 inflammasome activation. Furthermore, the HCK-specific inhibitor, A419259, attenuated the release of IL-1*β* and caspase 1(P20) from the macrophages and microglia and reduced the formation of the apoptosis-associated speck-like protein with a CARD domain (ASC) oligomer. We also observed that HCK binds to full length NLRP3 and its NBD(NACHT) and LRR domains, but not to the PYD domain. *In vivo*, the HCK inhibitor attenuated the LPS-induced inflammatory response in the liver of LPS-challenged mice. Collectively, these results suggested that HCK plays a critical role in NLRP3 inflammasome activation. Our results will enhance current understanding regarding the effectiveness of HCK inhibitors for treating acute inflammatory diseases.

## Introduction

The Nod-like receptor family protein 3 (NLRP3) inflammasome is a cytosolic protein complex composed of NLRP3, the adaptor protein apoptosis-associated speck-like protein with a CARD domain (ASC), and caspase 1, which are rapidly assembled in response to both infection with pathogens and endogenous “danger signals” such as monosodium urate, alum, silica, reactive oxygen species, amyloid *β*, and cholesterol (Duewell et al., 2010; Spreafico et al., 2010; Tschopp and Schroder, 2010; Peeters et al., 2013; Cho et al., 2014; Shaw et al., 2014; Kong et al., 2017). Activation of the NLRP3 inflammasome promotes the maturation and release of several proinflammatory cytokines, including interleukin-1*β* (IL-1*β*) and IL-18. Extracellular secretion of IL-1*β* and IL-18 requires two distinct signals: the signal driven by pattern recognition receptors, which induces the expression of pro-IL-1*β* and pro-IL-18 mRNAs (signal 1) and activation of the inflammasome (signal 2), which stimulates the cleavage of caspase 1 (Davis and Ting, 2010; Schroder and Tschopp, 2010; Lamkanfi and Dixit, 2012). Many studies have shown that the NLRP3 inflammasome is involved in diseases such as type 2 diabetes, atherosclerosis, colitis, Parkinson’s disease, and multiple sclerosis, as well as psychological disorders (Guarda et al., 2009; Duewell et al., 2010; Lamkanfi and Dixit, 2012; Heneka et al., 2013; Zhou et al., 2016).

Various protein kinases, including PAK1, PKA, PKC, PKD, PKR, BTK, PyK2, IRAK, Syk, and JNK1 have been reported to be required for inflammasome activation (Basak et al., 2005; Lu et al., 2012; Ito et al., 2015; Chung et al., 2016; Spalinger et al., 2016; Swanson and Ting, 2016; Stutz et al., 2017; Magnotti et al., 2019). A protein tyrosine phosphatase, non-receptor type 22 (PTPN22), interacts with and dephosphorylates NLRP3 at tyrosine 861, thereby activating the inflammasome (Spalinger et al., 2016). Phosphatase PP2A dephosphorylates NLRP3 at serine 295 and negatively regulates its activity (Stutz et al., 2017). Interestingly, phosphorylation of NLRP3 at serine 295 by different upstream kinases differentially regulates NLRP3 inflammasome activation. PKA-induced NLRP3 phosphorylation at serine 295 significantly blocks nigericin-induced inflammasome activation, although PKD-induced serine 295 phosphorylation at the Golgi apparatus is required for inflammasome activation. In addition to NLRP3, phosphorylation of ASC and caspase 1 is also involved in NLRP3 activation. Studies have shown that Syk is involved in the tyrosine phosphorylation of murine ASC, which promotes ASC speck formation and NLRP3/AIM2 activation (Chung et al., 2016). In addition, Pyk2, BTK, IKK*α*, and IKKi also participate in inflammasome activation by phosphorylating ASC (Martin et al., 2014). Overall, phosphorylation of NLRP3 by specific kinases diversely regulates inflammasome activation in the presence of different stimuli, which controls the temporal and spatial activation of the NRLP3 inflammasome.

The hematopoietic cell kinase (HCK), a member of the Src family of non-receptor tyrosine kinases (NRTKs), is primarily expressed in myeloid cells. HCK is highly expressed in macrophages, which is further augmented during macrophage activation. HCK is involved in diverse inflammatory responses (English et al., 1993). Knocking down of endogenous *Hck* in the murine macrophage cell line BAC1.2F5 interferes with the lipopolysaccharide (LPS)-induced *Tnfa* expression, without altering TNF-α release (Scholz et al., 2000). Furthermore, A419259, a specific pharmacological inhibitor of HCK, ameliorated bone destruction associated with inflammation (Kim et al., 2020). Therefore, we speculated that HCK might also be involved in the regulation of microglia (resident macrophages of the central nervous system) function and progression of Alzheimer’s disease-like neuropathology (Lim et al., 2018). However, the underlying mechanism is not known.

Hence, in this study, we aimed to investigate the role of HCK in NLRP3 inflammasome activation. HCK physically interacted with NLRP3, leading to the induction of ASC oligomerization and caspase- 1 activation in a kinase activity-dependent manner *in vitro*. We also observed that endogenous inhibition of HCK activity alleviated LPS-induced inflammatory response in the liver. These results might enhance our understanding regarding the mechanism of NLRP3 inflammasome activation and the effectiveness of HCK inhibitors for treating acute inflammatory diseases.

## Materials and Methods

### Mice

C57BL/6J mice were purchased from Charles River Co., Ltd. Male mice were used at about 8 weeks of age. All animal experiments were approved by and conformed to the guidelines of the institutional animal care and use committee of the Beijing Institute of Basic Medical Sciences.

### Cell Preparation and Culture

HEK293T cells were maintained in basic high glucose (Gibco) Dulbecco’s modified Eagle’s medium (DMEM) supplemented with 10% fetal bovine serum (FBS) (BI) and 1% penicillin/streptomycin (Gibco). Bone marrow-derived macrophages (BMDMs) were prepared as described previously (Oh et al., 2019) after sacrificing and immersing mice in 75% ethanol for 5 min. The mid-back skin was clipped and removed from the lower part of the body, and the tissue remaining on the pelvic and femoral bones was cleaned. The end of each bone was cut. The bone marrow was expelled from both ends of the bone using a 1 ml syringe filled with bone marrow growth medium. The cells were centrifuged at 350*g* for 5 min, and the red blood cells were lysed in ammonium chloride-potassium (ACK) buffer (0.154 M NH_4_Cl, 10 mM KHCO_3_, 0.1 mM EDTA) for 3 min. Subsequently, the cells were centrifuged at 350*g* for 5 min and resuspended in complete basic high glucose DMEM supplemented with macrophage colony stimulating factor (MCSF) (20 ng/ml) (Peprotech). The medium was changed with fresh MCSF-supplemented medium after every 3 days. The cells were ready for use in a week and were cultured for 3 weeks.

Murine peritoneal macrophages (PMs) were prepared as below. Briefly, mice were sacrificed and then immersed in 75% ethanol for 5 min. Five milliliters Roswell Park Memorial Institute (RPMI)-1640 medium (Gibco) was injected into the abdominal cavities after clipping the abdominal skin and exposing the abdomens. Next, the mice were shaken mildly to extract the liquid from the abdominal cavities. The collected cells were centrifuged at 350 × *g* for 5 min, and the re-suspended cells were cultured and maintained in complete RPMI-1640 medium (supplemented with heat-inactivated 10% FBS and 1% penicillin/streptomycin).

Microglia were prepared from the cortex of mice at the P0 stage as described previously (Cheng et al., 2017; Wu et al., 2019). The microglia were maintained in basic high glucose DMEM supplemented with heat-inactivated 20% FBS and 1% penicillin/streptomycin (Gibco).

### Regents and Antibodies

LPS (L2630) and ATP (A7699) were purchased from Sigma-Aldrich. Nigericin (HY-127019), JANEX-1 (HY-15508), and A419259 (HY-15764) were from MedChemExpress (Monmouth, NJ, USA). Vigofect (T001) was from Vigorous (Beijing, China). The Lipofectamine™ RNAiMAX transfection reagent was from ThermoFisher (Waltham, MA, USA). The anti-rabbit ASC antibody (1:1000; #67824) was from Cell Signaling Technology (Cambridge, MA, USA). The anti-mouse NLRP3 (1:1,000; AG-20B-0014) and anti-mouse caspase 1 (p20) (1:1,000; AG-20B-0042) antibodies were from AdipoGen (San Diego, CA, USA). The anti-mouse IL-1*β* antibody (1:1,000; AF-401-NA) was from R&D Systems (Minneapolis, MN, USA). The anti-rabbit HCK antibody (1:500; A14537) was from Abclonal (Wuhan, China) and anti-rabbit HCK antibody (1:200; 11600-1-AP) was from ProteinTech (Wuhan, China). Anti-mouse *β*-tubulin (1:2,000; CW0098A), anti-mouse GAPDH (1:3000; CW0100M), goat-anti mouse horse radish peroxidase (HRP) IgG(H + L) (1:5,000; CW0110S), and goat anti-rabbit HRP IgG (H + L) (1:5,000; CW0103S) were from CWBiotech (Beijing, China). The anti-mouse FLAG antibody (1:1,000; F1804) was from Sigma-Aldrich and anti-mouse MYC antibody (M047-3) was from MBL (Woburn, MA, USA). Goat anti-mouse IgG HRP (L) and goat anti-mouse HRP(Fc) were from Invitrogen.

### CellTiter-Glo^®^ Luminescent Cell Viability Assay

PMs were seeded in a 96-well plate (1 × 10^4^ cells/well), and A419259 (0.01, 0.1, 0.25, 0.5, and 1 μM) was added to the medium after 12 h. After 4 h, 100 μl of the CellTiter-Glo^®^ luminescent (G7570, Promega, USA) solution was added to the 96-well plate and mixed gently on an orbital shaker at room temperature for 10 min. The resulting fluorescence was measured using a multiscan spectrophotometer (TECAN, SPARK 10M).

### Inflammasome Activation Assays

BMDMs were seeded at the density of 1–2 × 10^5^ cells/well in 96-well plates, iBMDMs at 1 × 10^6^ cells/well in 6-well plates, PMs at 1 × 10^6^ cells/well in 24-well plates or 5 × 10^6^ cells/well 6-well plates, respectively, and THP1 cells at 1 × 10^6^ cells/well in 12-well plates. The following day, the medium was replaced, and cells were stimulated with 500 ng/ml LPS for 3 h. The medium was removed and replaced with serum-free medium containing dimethyl sulfoxide (DMSO; 1:1,000) and 1 μM A419259 and incubated for 1 h. The cells were then stimulated with the following inflammasome activators: 2.5 mM ATP (30–45 min) and 5 μM nigericin (30–45 min).

### Plasmids

3×FLAG-tagged ASC, 3×FLAG-tagged NLRP3, 3×FLAG-tagged PYD, 3×FLAG-tagged NBD, 3×FLAG-tagged LRRs, and MYC-tagged HCK were subcloned into the pCMV10 expression vector.

### SiRNA-Mediated Gene Silencing

BMDMs were planted in 96-well plates (1 × 10^5^ cells/ml) and THP1 cells were planted in 12-well plates (5 × 10^5^ cells/ml). The overnight medium was replaced after 12 h, and 40 nM siRNA was added to each well along with the Lipofectamine™ RNAiMAX transfection reagent according to the manufacturer’s guidelines. All the siRNAs were from GenePharma (Shanghai, China). The sequences used for HCK knockdown are shown in **Table 1**.

**Table 1:** SiRNA target sequences.

Name	Target Sequence (5′–3′)
*siHck1* *siHck2*	GGAGCCCAGCAUGUUGC CAAAGGUGGGCCGUUCC

### Transfection and Immunoprecipitation

The HEK293T cells were transfected with the plasmids expressing FLAG-NLRP3, FLAG-ASC, or MYC-HCK by using Vigofect. After 36 h, the cells were collected and resuspended in lysis buffer (25 mM Tris pH 7.4, 150 mM NaCl, 1 mM EDTA, 1% Nonidet-P40, 5% glycerol, protease inhibitor cocktail, and phosphatase inhibitor) at 4°C for 30 min. Then, the cell lysates were centrifuged at 20,000g at 4°C for 5 min. The supernatants were incubated with Pierce™ protein A/G magnetic beads (ThemoFisher) (incubated with 1 µg anti-mouse FLAG antibody or anti-mouse MYC antibody for 1h at room temperature) overnight at 4°C. The supernatants were discarded and the magnetic beads were washed thrice with lysis buffer. Then, each sample was dissolved in 60 μl 1× sodium dodecyl sulfate (SDS) loading buffer and heated in a 100°C dry heater for 10 min.

LPS-primed iBMDMs (5 × 10^6^ cells/well) (treated or not treated with 1 μM A419259 and stimulated with 5 μM nigericin for 1 h) were lysed with lysis buffer at 4°C for 30 min in 6 cm plate. Then, the cell lysates were centrifuged at 20,000g at 4°C for 5 min. The supernatants were incubated with Pierce™ protein A/G magnetic each (ThemoFisher) (incubated with 1 μg anti-HCK or 1 μg anti-ASC antibody for 1 h at room temperature) overnight at 4°C. The supernatants were discarded and the magnetic beads were washed thrice with lysis buffer. Then, the samples were dissolved in 60 μl 1× SDS loading buffer.

### Cross-Linking of the ASC Oligomer

LPS-primed PMs (5 × 10^6^ cells/well) were treated with 1 μM A419259 and stimulated with 5 μM nigericin for 30 min in 6-well plate. The cells were lysed with 0.5 ml lysis buffer (50 mM Tris-HCl pH 7.6, 0.5% Triton X-100) for 20 min and centrifuged at 6,000g for 15 min. The pellets were washed thrice with Tris-buffered saline (TBS) and resuspended in TBS containing 2 mM disuccinimidyl suberate (DDS) for cross-linking at 37°C for 45 min. Next, the pellets were centrifuged at 6,000 × *g* for 15 min and dissolved in 50 μl 1× SDS loading buffer.

### Immunoblotting

Cells were lysed using the cell lysis buffer used for immunoprecipitation and then boiled in × SDS loading buffer. The cell culture supernatants were concentrated using methanol/chloroform and the pellets were lysed in the SDS sample buffer. Equal amounts of total protein were subjected to SDS-polyacrylamide gel electrophoresis (PAGE) analysis, and immunoblotting with the appropriate antibodies was performed as described previously. For immunoblotting, the nitrocellulose blot with the transferred proteins was incubated with HRP-conjugated antibody in 5% skim milk-TBS-Tween 20 (0.1%) for 1 h with shaking. After three washes, the antigen–antibody complexes were visualized using enhanced chemiluminescence (ECL) reagents.

### Enzyme-Linked Immunosorbent Assay (ELISA)

Supernatants from cell culture or serum were assayed for mouse TNF-α (AF-410-NA, R&D Systems) and IL-1*β* (MLB00C, R&D Systems) levels according to the manufacturer’s instructions.

### Quantitative Reverse Transcription-Polymerase Chain Reaction (qRT-PCR)

Total RNA was extracted using the Trizol reagent (Invitrogen). cDNA was obtained using *TransScript* ^®^II One-Step gDNA removal and cDNA synthesis super mix (TransGen Biotech, Beijing, China), following the manufacturer’s instructions. QRT-PCR analysis was performed in a LightCycler 480 (Roche) using specific primers, 40 ng cDNA, and the Ultra SYBR master mix (HighROX) (Cwbiotech). Relative mRNA expression was obtained using the ΔΔCt method. *Actb* or *Gapdh* was used as reference genes. The primers used for murine genes are shown in **Table 2**.

**Table 2:** Primers for RT-qPCR.

Name	Sequence (5′–3′)
*Nlrp3-*forward *Nlrp3-*reverse *Caspase1*-forward *Caspase1*-reverse *Il1b-*forward *Il1b-*reverse *Il-18*-forward *Il-18*-reverse *Asc*-forward *Asc*-reverse *Tnf-a-*forward *Tnf-a-*reverse *Il6-*forward *Il6-*reverse *Il10-*forward *Il10-*reverse *Il4-*forward *Il4-*reverse *Hck-*forward *Hck-reverse* *Actb-*forward *Actb-*reverse *Gapdh-*forward *Gapdh-*reverse	ATTACCCGCCCGAGAAAGG TCGCAGCAAAGATCCACACAG ACAAGGCACGGGACCTATG TCCCAGTCAGTCCTGGAAATG GCAACT GTTCCTGAACTCAACT ATCTTTTGGGGTCCGTCAACT GACTCTTGCGTCAACTTCAAGG CAGGCTGTCTTTTGTCAACGA CTTGTCAGGGGATGAACTCAAAA GCCATACGACTCCAGATAGTAGC CCCTCACACTCAGATCATCTTCT GCTACGACGTGGGCTACAG CCAAGAGGTGAGTGCCTTCCC CTGTTGTTCAGACTCTCTCCCT GCTCTTACTGACTGGCATGAG CGCAGCTCTAGGA GCATGTG GGTCTCAACCCCCAGCTAGT GCCGATGATCTCTCTCAAGTGAT TCCTCCGAGATGGAAGCAAG ACAGTGCGACCACAATGGTAT GGCTGTATTCCCCTCCATC CCAGTTGGTAACAATGCCATGT AGGTCGGTGTGAACGGATTTG TGTAGACCATGTAGTTGAGGTCA

### Immunofluorescence

Murine PMs (5 × 10^5^ cells/well) were seeded on coverslips coated with 1× poly-L-lysine-ornithine in 24-well plate. LPS-primed murine PMs were treated with 1 μM A419259 for 1 h, stimulated with 1 mM nigericin for 30 min, and then fixed and permeabilized using 4% formaldehyde and 0.5% Triton X-100. The cells were incubated overnight with anti-rabbit ASC antibody (1:500; CST). Next, the cells were incubated with Alexa Fluor 488-conjugated anti-rabbit IgG antibody (1:400). Nuclei were counterstained with DAPI. The cells were examined under a laser scanning confocal microscope (Nikon A1).

### *In Vivo* LPS Challenge

C57BL/6 mice were injected intraperitoneally (i.p.) with 30 mg/kg A419259 or the vehicle control (DMSO/PBS) 24 h 12 h before i.p. injection of 5 mg/kg LPS or PBS. Mice were sacrificed after 3 h, and the tissues were stored at −80°C and prepared for immunoblotting.

### Statistical Analysis

All values were expressed as the mean ± SEM. All statistical analyses were performed using GraphPad Prism version 6.0 software. The significance of differences was assessed by unpaired Student’s t test or one-way or two-way analysis of variance (ANOVA) followed by Tukey’s multiple comparisons test as indicated. P value < 0.05 was considered significant. Error bars represent SEM.

## Results

### HCK Is an Upstream Regulator of the NLRP3 Inflammasome

**Figure 1:**
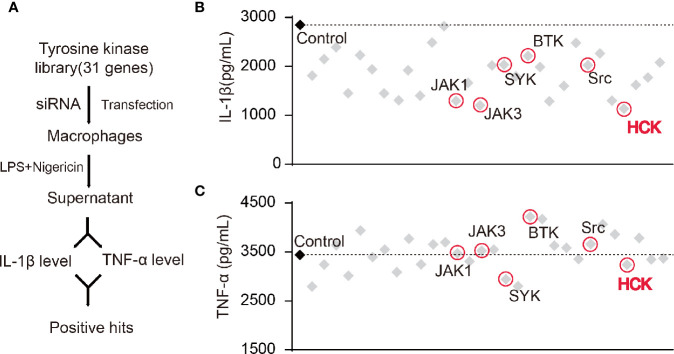
Screening of HCK as a regulator for the NLRP3 inflammasome. **(A)** Flowchart showing the candidate siRNA library targeting 31 putative tyrosine kinases in BMDM. IL-1*β* and TNF-α levels were determined. **(B, C)** Enzyme-linked immunosorbent assay (ELISA) for IL-1*β* **(B)** or TNF-α **(C)** in supernatants from LPS-primed (500 ng, 3 h) PMs transfected with control siRNA (scrambled) or siRNAs specific for the individual tyrosine kinases post-nigericin (5 μM) stimulation for 30 min.

Studies have shown that certain NRTKs are involved in the activation of the NLRP3 inflammasome. Hence, we constructed a NRTK siRNA library and designed a screening process (**Figure 1A**). We transfected macrophages with siRNAs, and the supernatants were collected 72 h after LPS and nigericin (a NLRP3 activator) stimulation; the levels of secreted IL-1*β* and TNF-α were detected using ELISA. The results showed that the knockdown of *Hck* and Janus protein tyrosine kinase 1 (*Jak1*) and *Jak3* significantly reduced the levels of IL-1*β* in the supernatant (**Figures 1B, C**). However, production of TNF-α, an inflammasome-independent cytokine, was not affected, suggesting that knocking down of *Hck*, *Jak1*, and *Jak3* inhibited the release of IL-1*β via* inflammasome activation.

### The HCK Inhibitor Attenuated NLRP3 Inflammasome-Induced Inflammatory Response in Macrophages and Microglia

**Figure 2:**
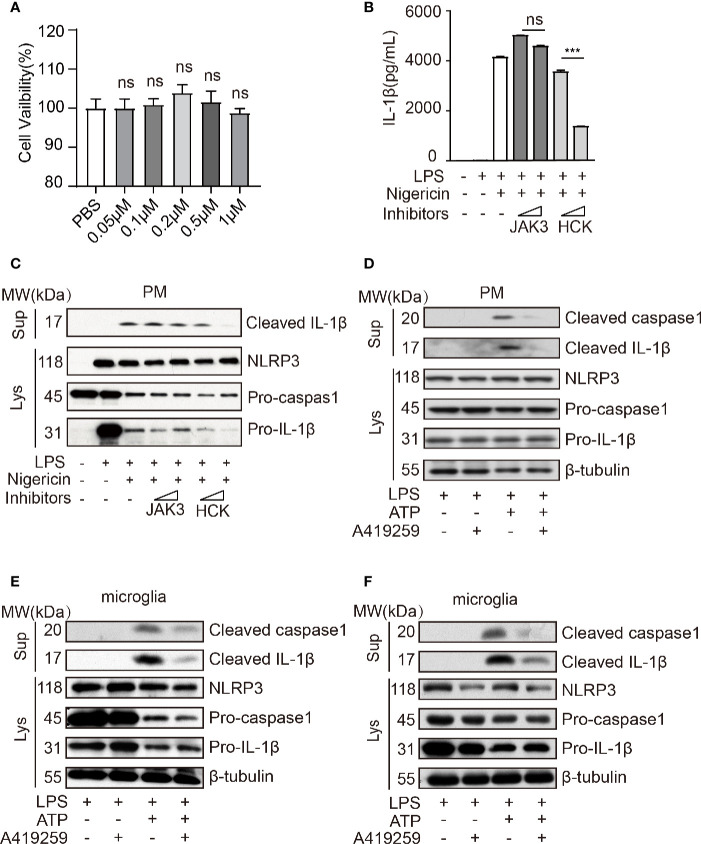
HCK inhibition attenuated NLRP3 inflammasome-induced inflammatory response in macrophages and microglia. **(A)** PMs were seeded in a 96-well plate (1 × 10^4^ cells/well) plate. After 12 h, the cells were treated with A419259 for 4 h, and cell viability was detected. **(B)** ELISA for cleaved IL-1*β* (P17) in the supernatants of LPS-primed (500 ng, 2 h) PMs treated with JAK3(JANEX-1) or HCK(A419259) inhibitor for 1 h and stimulated with nigericin for 30 min. **(C)** Immunoblotting analysis of cleaved IL-1*β* (P17) in the supernatants from LPS-primed (500 ng, 2 h) PMs treated with JAK3(JANEX-1) or HCK(A419259) inhibitor for 1 h and stimulated with nigericin for 30 min, followed by immunoblotting for pro-IL-1*β* or NLRP3 antibody. **(D)** Immunoblotting analysis of cleaved IL-1*β* (P17) and cleaved caspase 1 (P20) in the supernatants of LPS-primed (500 ng, 2 h) PMs treated for 1 h with the HCK inhibitor (A419259) and then stimulated with 1.5 mM ATP for 30 min, followed by immunoblotting analysis with pro-IL-1*β*, pro-caspase 1, NLRP3, or *β*-tubulin antibodies in whole cell lysates. **(E, F)** Immunoblotting analysis of cleaved IL-1*β* or cleaved caspase 1 in the supernatants of LPS-primed (500 ng, 2 h) microglia treated for 1 h with HCK inhibitors (A419259) and then stimulated with 5 μM nigericin **(E)** or 1.5 mM ATP **(F)** for 30 min. The cell lysates were immunoblotted with antibodies against pro-IL-1*β*, pro-caspase 1, NLRP3, or *β*- tubulin in cell lysates. Data from **(A**–**F)** are representative of at least three independent experiments. Data show means ± SEM. ***P < 0.001, ns, *P* > 0.05, Student’s *t*-test.

Wang et al. has reported that *Jak1* knockdown blocked the phosphorylation of NF-*κ*B p65 in the nuclei and that of NF-*κ*B I*κ*B*α* in the cytoplasm and attenuated NLRP3 inflammasome activation (Wang et al., 2017). A previous study has shown that HCK, NF-*κ*B signaling, and JAK are triggered by the *Myd88* mutant (kinase activation mutant) in B cells (Ngo et al., 2011; Yang et al., 2016), and MYD88 acts upstream of NLRP3. Hence, to further confirm these results, we used JAK3 inhibitor (JANEX-1) and HCK inhibitor (A419259, the Src family kinases specific inhibitor) to verify whether HCK and JAK3 were involved in the regulation of NLRP3 inflammasome activation. Interestingly, we observed that HCK inhibition, but not JAK3 inhibition, significantly reduced LPS/nigericin-mediated upregulation of IL-1*β* secretion in the cell culture supernatants, and A419259 did not affect the cell viability (**Figures 2A–C**). Intriguingly, A419259 did not inhibit the LPS-induced IL-1*β* and NLRP3 expression (**Supplementary Figure 1A**). In addition to nigericin, the NLRP3 inflammasome can be also activated by ATP, silica, monosodium urate, and calcium (Dostert et al., 2008; Franchi et al., 2009; He et al., 2012; Rossol et al., 2012; He et al., 2016). Hence, we selected ATP as another secondary signaling stimulation to confirm the results obtained using nigericin. As expected, ATP treatment of LPS-primed macrophages increased intracellular cleaved caspase 1 expression and the level of secreted IL-1*β* in the culture medium, both of which were considerably attenuated by the HCK inhibitor, A419259 (**Figure 2D**). The protein levels of NLRP3, pro-caspase 1, and pro-IL-1*β* were not significantly affected (**Figure 2D**).

Furthermore, deficiency of HCK in PMs could suppress the IL-1*β* secretion and caspase1 activation without affecting the expression levels of NLRP3, pro-IL-1*β* and caspase1 (**Supplementary Figures 2A, B**). We also transfected the human monocyte cell line, THP1, with *Hck*-specific siRNA or mock control and observed that *Hck* knockdown significantly decreased IL-1*β* secretion in the LPS-primed THP-1 cells treated with nigericin without altering the expression levels of NLRP3 and pro-IL-1*β* (**Supplementary Figure 3A**). The HCK inhibitor also attenuated the secretion of IL-1*β* in the culture medium of nigericin-stimulated LPS-primed THP1 (**Supplementary Figure 3B**).

The microglia perform functions similar to those of macrophages in the central nervous system. Hence, we speculated that A419259 might also inhibit NLRP3 inflammasome activation in the microglia. We used nigericin and ATP as secondary signaling stimuli to activate the NLRP3 inflammasome. The results showed that secretion of cleaved IL-1*β* and cleaved caspase 1 from the A419259-treated group was lower than that from the group treated with LPS and nigericin or ATP (**Figures 2E, F** **)**.

Taken together, these results demonstrated that HCK was involved in NLRP3 inflammasome activation and subsequent IL-1*β* production, indicating that NLRP3 inflammasome activation depended on HCK kinase activity.

### HCK Inhibition Reduced Inflammasome Assembly

**Figure 3:**
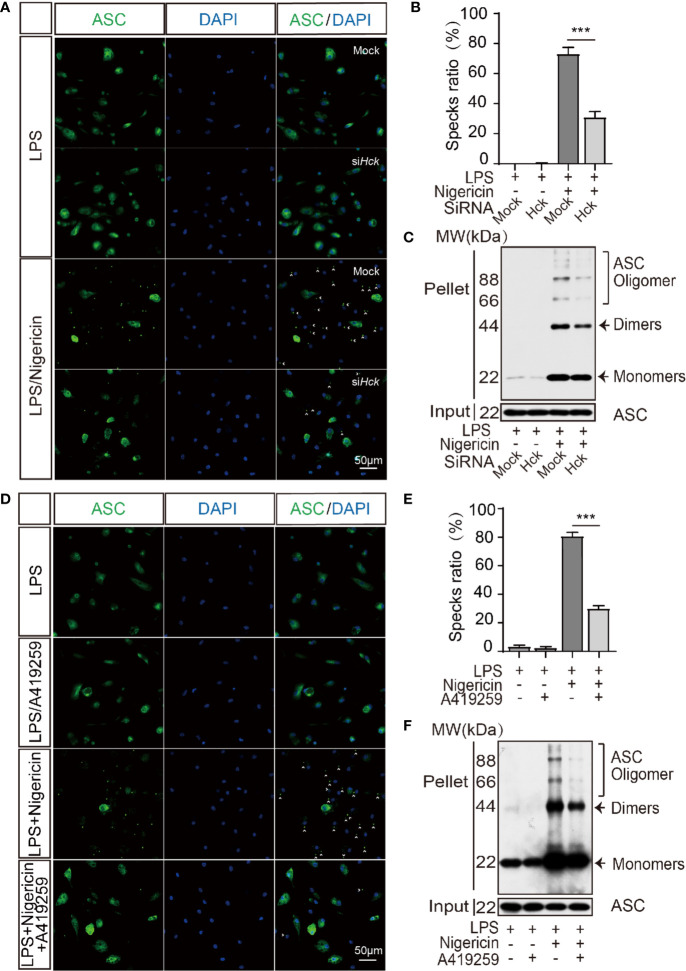
HCK promotes the oligomerization of ASC. **(A, B)** Immunostaining of ASC in the LPS-primed PMs transfected with control siRNA (Mock) or siHCK for 3 h, followed by stimulation with nigericin for 30 min. **(C)** Immunoblotting analysis of the purified cross-linked ASC oligomers from the whole cell lysates in the LPS-primed (500 ng, 3 h) PMs transfected with control siRNA (Mock) or siHCK for 72 h, followed by stimulation with 5 μM nigericin for another 30 min **(D, E)** Immunostaining of ASC in the LPS-primed PMs after 1 h-incubation with A419259, followed by stimulation with Nigericin for 30 min. Nuclei were counterstained with DAPI. Fluorescence was imaged using confocal microscopy. **(F)** Immunoblotting analysis of the purified cross-linked ASC oligomers from the whole cell lysates in the LPS-primed (500 ng, 2 h) PMs treated with HCK inhibitor (A419259) for 1 h, followed by stimulation with 5 μM nigericin for another 30 min. Scale bar is 50 μm in all figures. Arrowheads denote ASC specks, and the percentage of ASC speck is shown in **(B**, **E)**. Data from **(A**–**F)** are representative of at least three independent experiments. Data show means ± SEM, ***P < 0.001, Student’s *t*-test.

Oligomerization of ASC is an initiating event of NLRP3 inflammasome activation and is responsible for the recruitment and activation of caspase 1. We speculated that ASC oligomerization might be affected by HCK. Hence, we analyzed ASC oligomerization *via* ASC specks staining and DSS-induced ASC cross-linking assay. The results showed that compared to the siCtrl cells, the number of ASC specks was decreased after inflammasome activation in PMs transfected with siHCK (**Figures 3A, B**). Furthermore, DSS-induced ASC oligomerization also showed decrease in HCK deficiency PMs (**Figure 3C**). In Addition, A419259 could suppress the ASC specks and DSS-induced ASC oligomerization formation as well (**Figures 3D–F**). These results indicated that deficiency of HCK and HCK kinase activity inhibition could reduce ASC oligomerization and inflammasome assembly. To further decipher the mechanism underlying HCK-mediated regulation of ASC oligomerization, we analyzed the interaction between HCK and ASC. We observed lack of direct interaction between ASC and HCK using reciprocal immunoprecipitation (**Supplementary Figures 4A–C**). However, HCK inhibition markedly mitigated the interaction between ASC and NLRP3, indicating that HCK might directly affect NLRP3 function during inflammasome activation (**Supplementary Figures 4C, D**).

### HCK Interacted With NLRP3 Independent of PYD

**Figure 4:**
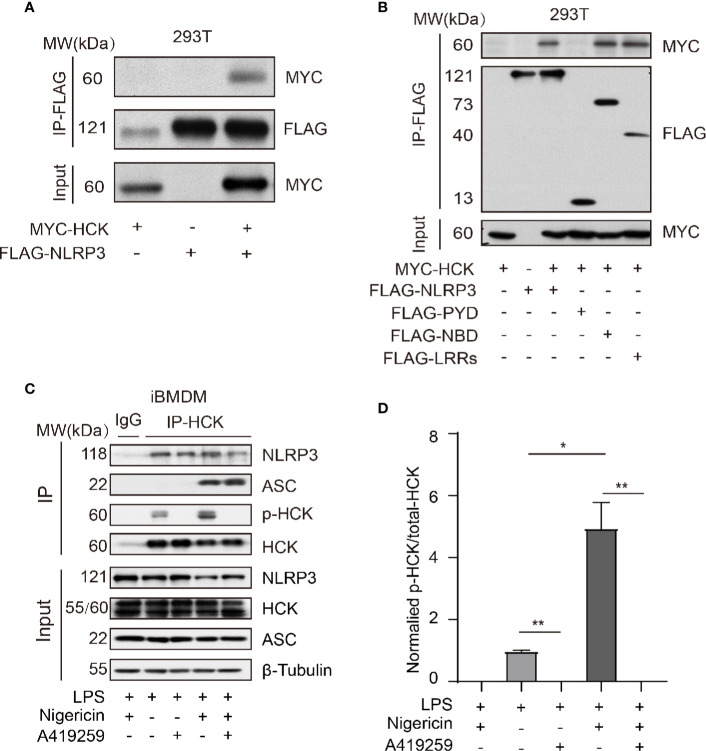
HCK interacts with NLRP3 independent of its PYD domain. **(A)** HEK293T cells were transfected with MYC-HCK (WT) and FLAG-NLRP3, and the interaction between HCK and NLRP3 was analyzed using immunoprecipitation (IP) with anti-FLAG antibody and immunoblotting (IB) with the anti-FLAG and anti-MYC antibodies. **(B)** HEK293T cells were transfected with MYC-HCK (WT) and FLAG-NLRP3 or its truncated forms (FLAG-PYD, FLAG-NBD, or FLAG-LRRs, followed by immunoprecipitation (IP) with anti-FLAG and immunoblotting (IB) with anti-FLAG or anti-MYC antibodies. **(C)** LPS-primed PMs were treated with A419259 and then stimulated with 5 μM nigericin for 30 min, followed by immunoprecipitation (IP) with HCK antibody and immunoblotting (IB) with antibodies against HCK, phosphorylated tyrosine, NLRP3, ASC or *β*-tubulin. **(D)** The normalized levels of HCK phosphorylation were quantified. Data from **(A**–**C)** are representative of at least three independent experiments. Data show means ± SEM, *P > 0.05, **P < 0.01, Student’s *t*-test.

Next, we aimed to investigate the mechanism underlying HCK-mediated regulation of NLRP3 inflammasome activation. The MYC-HCK, FLAG-NLRP3, and FLAG-ASC plasmids were transfected into HEK293T cells. The results showed that full length HCK bound to full length NLRP3 (**Figure 4A**). NLRP3 consists of three domains, namely, the PYD domain at the N-terminus, NBD (NACHT) domain in the middle, and LRRs at the C-terminus. To further characterize the domain of NLRP3 that interacts with HCK, we constructed plasmids harboring truncated NLRP3, including FLAG-PYD, FLAG-NBD, and FLAG-LRRs. Results of co-immunoprecipitation and immunoblotting experiments showed that HCK interacted with NBD and LRRs, but not PYD (**Figure 4B**). Furthermore, endogenous HCK interacted with NLRP3 in HEK293T cells. Interestingly, A419259 reduced the interaction between HCK and NLRP3 in iBMDMs (**Figure 4C**). We also observed that HCK phosphorylation increased when the NLRP3 inflammasome was activated by nigericin (**Figure 4D**). Overall, these results suggested that HCK could bind to NLRP3 *via* NBD and LRR domains.

### A419259 Reduced Inflammasome Activation *In Vivo* in Mice

**Figure 5:**
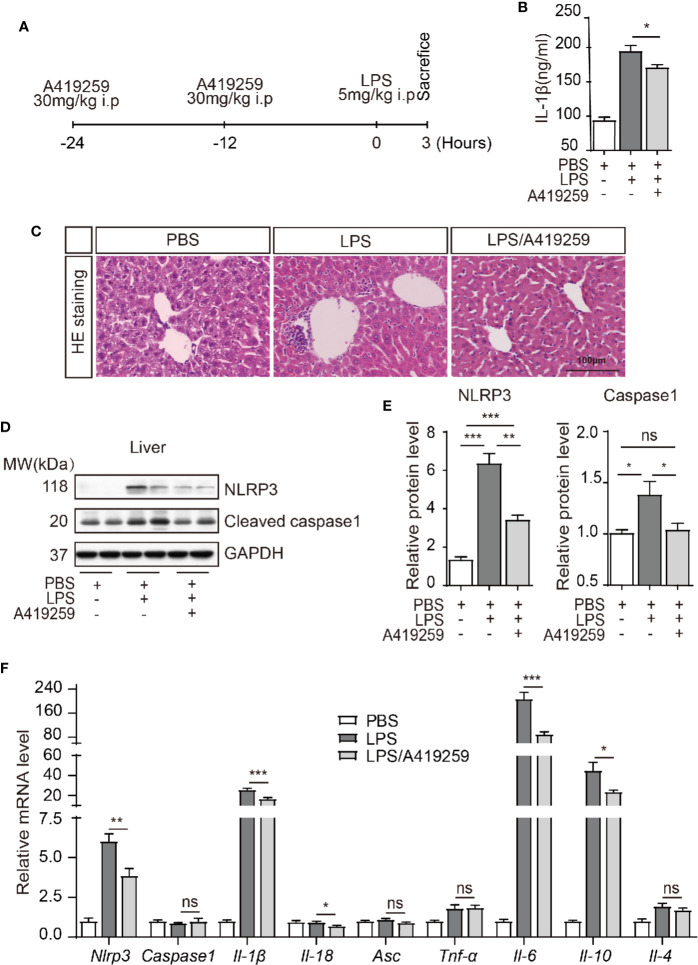
The HCK inhibitor A419259 attenuated LPS-induced inflammatory response *in vivo.* **(A)** Schematic graph illustrating the experimental timelines. **(B)** ELISA of the serum cleaved IL-1*β* (P17) from mice intraperitoneally injected with LPS (5 mg/kg of body weight) with or without A419259 (30 mg/kg of body weight) (n = 7 for each group). **(C)** Representative images of liver H&E staining (three mice, nine slices each group). **(D)** Immunoblotting analysis of cleaved caspase 1 (P20), NLRP3, or GAPDH in the livers of mice intraperitoneally injected with LPS with or without A419259 (n = 7 for each group). **(E)** Analysis of the changes in NLRP3 or cleaved caspase 1 (P20) protein level. **(F)** qRT-PCR analysis of *nlrp3, Caspase1, Il-1β, Il-18, Asc, Tnf-α, Il-6, Il-10*, and *Il-4* in the livers of mice intraperitoneally injected with LPS with or without A419259 (n = 7 for each group). Data show means ± SEM, *P < 0.05, **P < 0.01, ***P < 0.001. ns, *P* > 0.05, Student’s *t*-test.

HCK activation led to NLRP3 inflammasome complex assembly and activation. HCK was phosphorylated, which then bound to NLRP3, when the macrophages were stimulated with LPS (**Supplementary Figure 5**). In the presence of the secondary signal, HCK promoted NLRP3 inflammasome complex assembly and activation, and consequently, pro-IL-1*β* was cleaved by caspase 1 (P20) and secreted. To assess whether the effects of HCK on inflammasome activation *in vitro* could be translated *in vivo*, LPS-induced mouse models of inflammasome activation were used (**Figure 5A**). We evaluated serum IL-1*β* production in mice after i.p. injection of LPS and observed that LPS injection markedly increased serum IL-1*β* level, which was abolished by A419259 pre-treatment (**Figure 5B**). Consistent with our previous results, mice treated with A419259 showed reduced response to LPS, with lower amounts of secreted IL-1*β* than in vehicle-treated mice. We analyzed the liver damage *via* HE staining. A419259 could markedly decrease the inflammatory cells’ infiltration in the liver (**Figure 5C**). In addition, the protein levels of NLRP3 and caspase 1 (P20) in the liver decreased when the mice were treated with A419259 (**Figures 5D, E**). LPS treatment induced strong inflammatory response, as was evident from the increase in the levels of *Nlrp3*, *Il-1β, Il-6*, and *Il-10* mRNAs in the liver, which were mitigated by A419259 treatment (**Figure 5F**). However, *Caspas1, Asc, Tnf-a*, and *Il-4* expression did not differ between vehicle-treated and LPS-treated mice (**Figure 5F**). Collectively, these results confirmed the involvement of HCK during inflammasome activation *in vivo*, and showed that the HCK kinase inhibitor, A419259, attenuated LPS-induced inflammatory response in the liver.

## Discussion

NRTKs have been shown to play important roles in NLRP3 inflammasome activation. However, the functions and regulatory mechanisms of NRTKs during NLRP3 inflammasome activation are still not clear. Ito et al. showed that the tyrosine kinase BTK promoted ASC oligomerization by interacting with ASC and NLRP3 and activated the NLRP3 inflammasome (Ito et al., 2015). However, Mao et al. revealed that BTK deficiency augmented NLRP3 inflammasome activation by inhibiting the PP2A-mediated dephosphorylation of serine 5 in the pyrin domain of NLRP3 (Mao et al., 2020). Hence, regulation of NLRP3 phosphorylation requires further investigations. In this study, we identified a new tyrosine kinase, HCK, which activated the NLRP3 inflammasome *via* interaction with NLRP3 and promotion of ASC oligomerization.

Post-translational modifications of the NLRP3 complex include phosphorylation, acetylation, ubiquitination, oligomerization, and proteolytic cleavage, among which phosphorylation has been studied extensively. Phosphorylation mediated by diverse protein kinases has been reported to be involved in NLRP3 inflammasome activation. The protein tyrosine phosphatase, PTPN22, dephosphorylates NLRP3 *via* direct interaction with NLRP3 Tyr861 and induces NLRP3 inflammasome activation (Spalinger et al., 2016). The phosphatase PP2A is involved in the dephosphorylation of Ser5 and negatively regulates NLRP3 inflammasome activity (Martin et al., 2014). Serine 295 phosphorylation of NLRP3 performs dual functions with respect to NLRP3 inflammasome activation. PKA-induced Ser295 phosphorylation in human significantly blocks nigericin-induced inflammasome activation; however, PKD-induced Ser295 phosphorylation at the Golgi apparatus is required for inflammasome activation (Guo et al., 2016). Hara and Tsuchiya showed that signaling pathway-dependent kinases, Syk and JNK, activate the NLRP3 inflammasome by activating caspase 1 [13].

HCK of the NRTK family is localized in the cytoplasm and executes its functions as a kinase. It is generally activated *via* binding with target proteins, which it subsequently phosphorylates. We speculate that HCK might directly phosphorylate NLRP3. However, we failed to detect the HCK-induced NLRP3 phosphorylation change by Phos-Tag analysis and phosphorylation mass-spectrometry (data not shown). Hara et al. have recently reported that tyrosine phosphorylation of human ASC-Y144 (corresponding to Y146 in mouse ASC) is required for inflammasome activation, and that phosphorylated ASC can be detected in the Triton X-insoluble fraction (Chung et al., 2016). We also found that HCK could not lead to tyrosine phosphorylation of ASC by using Phos-Tag analysis (data not shown). In this study, we observed that HCK regulated the activation of the NLRP3 inflammasome by interacting with NLRP3. We could not detect any interaction between HCK and ASC; however, the oligomerization of ASC in PMs decreased when the kinase activity of HCK was inhibited. HCK did not interact with ASC when the inflammasome was not activated. Therefore, we believe that HCK might indirectly promote ASC oligomerization. How HCK regulates NLRP3 activation post-binding warrants and the oligomerization of ASC need to further investigate.

Recent studies have shown that the activation of the NLRP3 inflammasome is involved in the progression of various inflammatory diseases, such as type 2 diabetes, atherosclerosis, and Muckle-Wells syndrome (Duewell et al., 2010; Lamkanfi and Dixit, 2012; Kong et al., 2017). Furthermore, the inflammasome is also reported to be involved in neurodegenerative diseases such as multiple sclerosis, Parkinson’s disease, and Alzheimer’s disease. Various studies have demonstrated that the anti-IL-1*β* antibody is effective in treating some of these disorders. HCK plays important roles in inflammatory response and is activated in tumor-associated immune cells. Genetic ablation of *Hck* in mice reduced cytokine expression in response to LPS stimulation and moderately increased the susceptibility of *Hck* knockout mice to infection (Poh et al., 2015). In this study, we observed that the HCK kinase activity inhibitor, A419259, restrained NLRP3 inflammasome activity in macrophages and microglia and attenuated the LPS-induced inflammatory response *in vivo*. Thus, A419259 might be a candidate drug for treating NLRP3 inflammasome activation-related diseases. Further investigations into the mechanism of HCK-mediated NLRP3 inflammasome activation are required to show HCK inhibition as a new strategy for the treatment of inflammatory disease.

## Data Availability Statement

The raw data supporting the conclusions of this article will be made available by the authors, without undue reservation.

## Ethics Statement

The animal study was reviewed and approved by Beijing Institute of Basic Medical Science.

## Author Contributions

XK, ZY, JC, and SW conceived and designed the project and drafted the manuscript. XK, YL, and LZ performed the experiments. All authors contributed to the article and approved the submitted version.

## Funding

This work was supported by grants from the National Nature Science Foundation of China (81671274 and 31600946 to SW; No. 81930029 to ZY; No.81400987 to JC; No. 81701187 to YL).

## Conflict of Interest

The authors declare that the research was conducted in the absence of any commercial or financial relationships that could be construed as a potential conflict of interest.
