# Supplementary material for: Hematopoietic Cell Kinase (HCK) Is Essential for NLRP3 Inflammasome Activation and Lipopolysaccharide-Induced Inflammatory Response In Vivo
Source: Front Pharmacol. 2020 Sep 15;11:581011. doi: 10.3389/fphar.2020.581011 (PMC7523510; doi:10.3389/fphar.2020.581011)
Supplement: Supplementary file 1 [file DataSheet_1.pdf]

Supplementary Material

Supplemental Figure 1

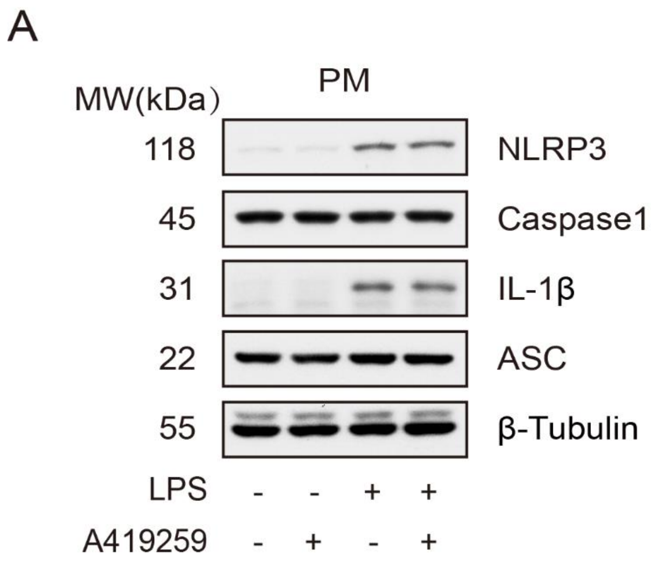

Supplementary Figure 1. A419259 did not inhibit the LPS-induced Pro-IL-β expression. (A) Immunoblotting analysis with ASC, IL-1β, Caspase-1, NLRP3 or β-tubulin antibodies in whole cell lysates that cells were treated with LPS (500ng) for 3h.

Supplemental Figure 2

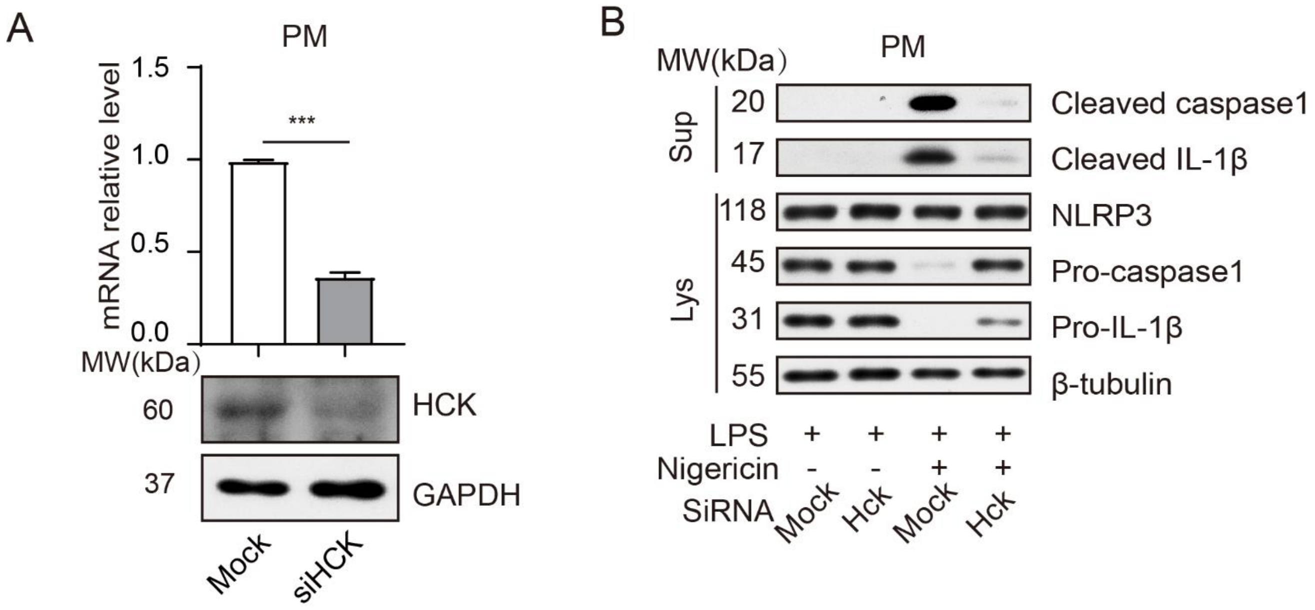

Supplementary Figure 2. Deficiency of HCK suppressed NLRP3 inflammasome activation in macrophages. (A) Immunoblotting and RT-PCR analysis of HCK in PMs transfected with control siRNA (Mock) or *Hck*-specific siRNA; (B) Immunoblotting analysis of cleaved IL-1β (P17) and Caspase1(P20)in the supernatants of LPS-primed (500 ng, 3 h) PM, which were transfected with control siRNA(Mock) or *Hck*-specific siRNA and stimulated with 5μM nigericin for 30 min. The cellular lysates were immunoblotted with antibodies against NLRP3, Caspase1, Pro-IL-1β or β-tubulin in whole cell lysates.

Supplemental Figure 3

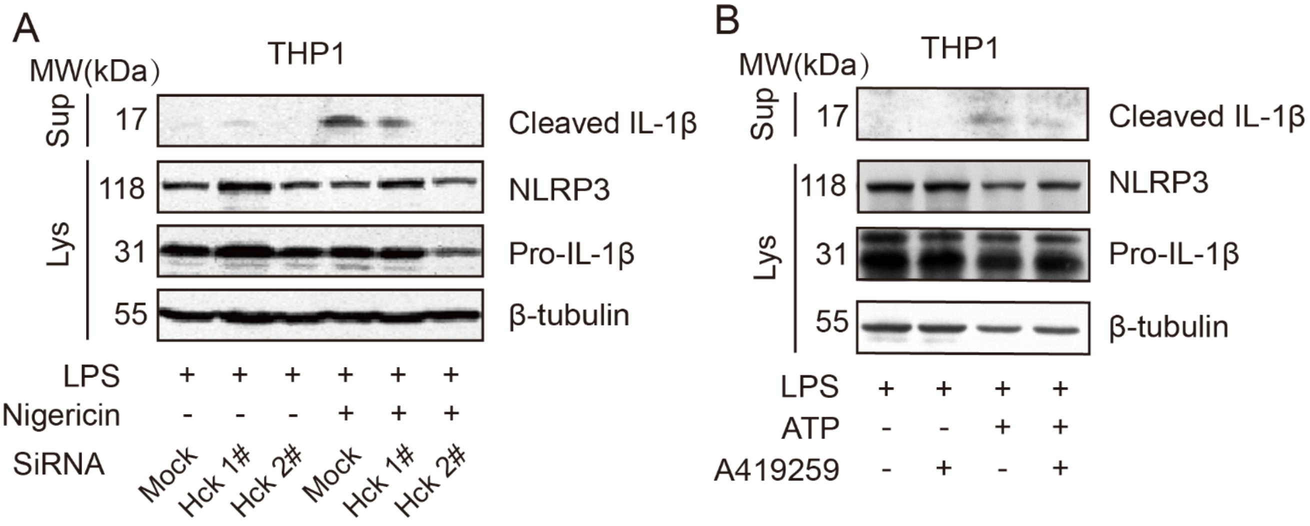

Supplementary Figure 3. A419259 attenuated NLRP3 inflammasome-induced inflammatory response in THP1. (A) Immunoblotting analysis of cleaved IL-1β (P17) in the supernatants of LPS-primed (500 ng, 3 h) THP1 cells, which were transfected with control siRNA or *Hck*-specific siRNA and stimulated with 5μM nigericin for 30 min. The cellular lysates were immunoblotted with antibodies against pro-IL-1β, NLRP3 or β-tubulin in whole cell lysates. (B) Immunoblotting analysis of cleaved IL-1β (P17) in the supernatants from LPS-primed THP1 treated for 1 h with the HCK inhibitor (A419259) and then stimulated with 1.5 mM ATP for 1 h, followed by immunoblotting analysis with antibodies against pro-IL-1β, NLRP3 and β-tubulin in whole cell lysates.

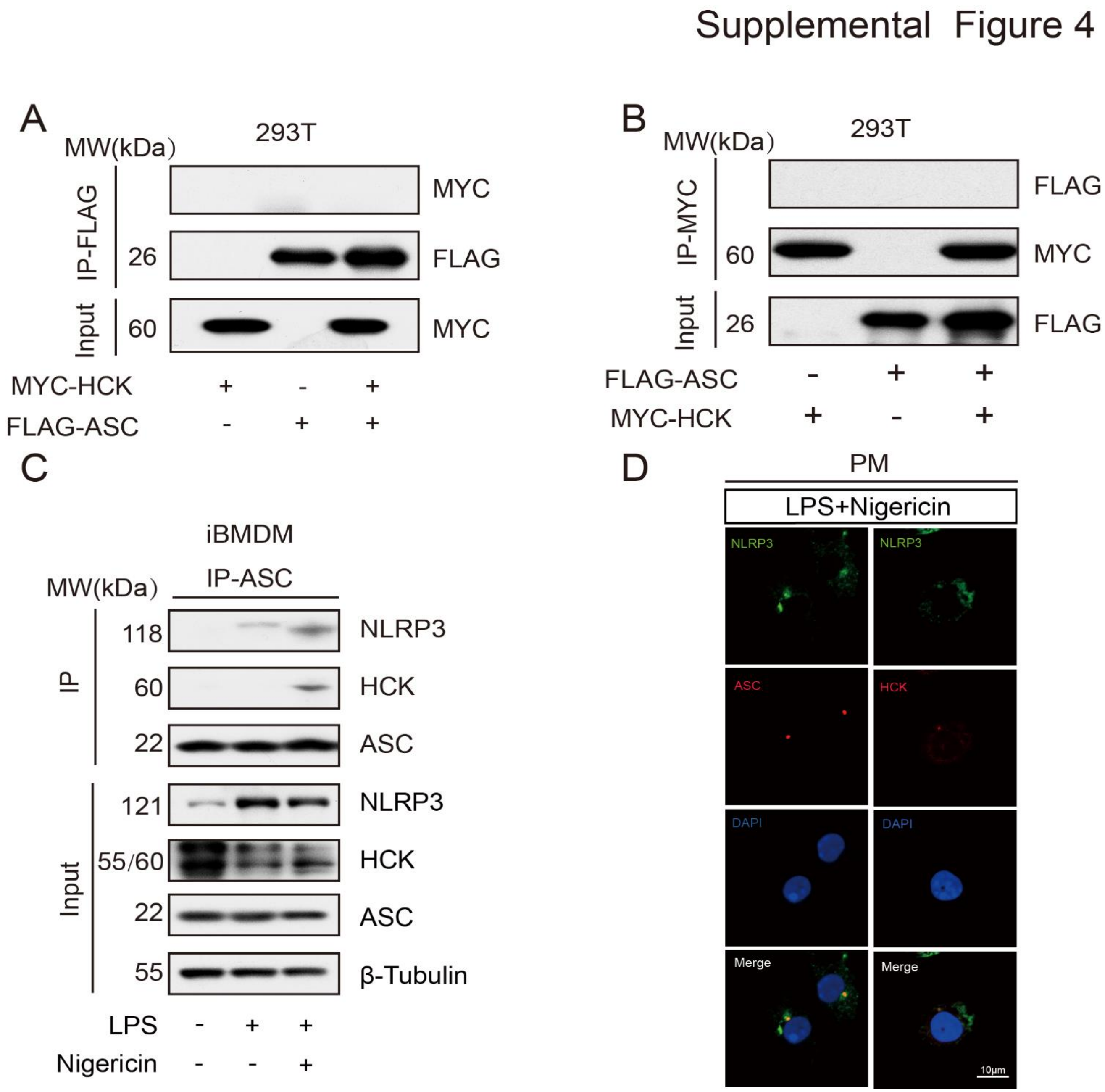

Supplementary Figure 4. HCK does not directly bind to ASC. (A) HEK293T cells were transfected with MYC-HCK and FLAG-ASC, and their interaction was analyzed using immunoprecipitation (IP) with anti-FLAG, followed by immunoblotting. (B) HEK293T cells were transfected with MYC-HCK and FLAG-ASC, and interaction was analyzed using immunoprecipitation (IP) with anti-MYC and immunoblotting. (C) LPS-primed iBMDMs were stimulated with 5 μM nigericin for 30 min, and interaction was analyzed using immunoprecipitation (IP) with anti-ASC antibody and immunoblotting (IB) with antibodies against NLRP3, HCK, ASC or β-tubulin. (D) Immunostaining of HCK and NLRP3 or ASC and NLRP3 in the PMs treated with LPS for 3h, followed by stimulation with nigericin for 30 min. Nuclei were counterstained with DAPI. Fluorescence was imaged using confocal microscopy. Data from A~C are representative of at least three independent experiment.

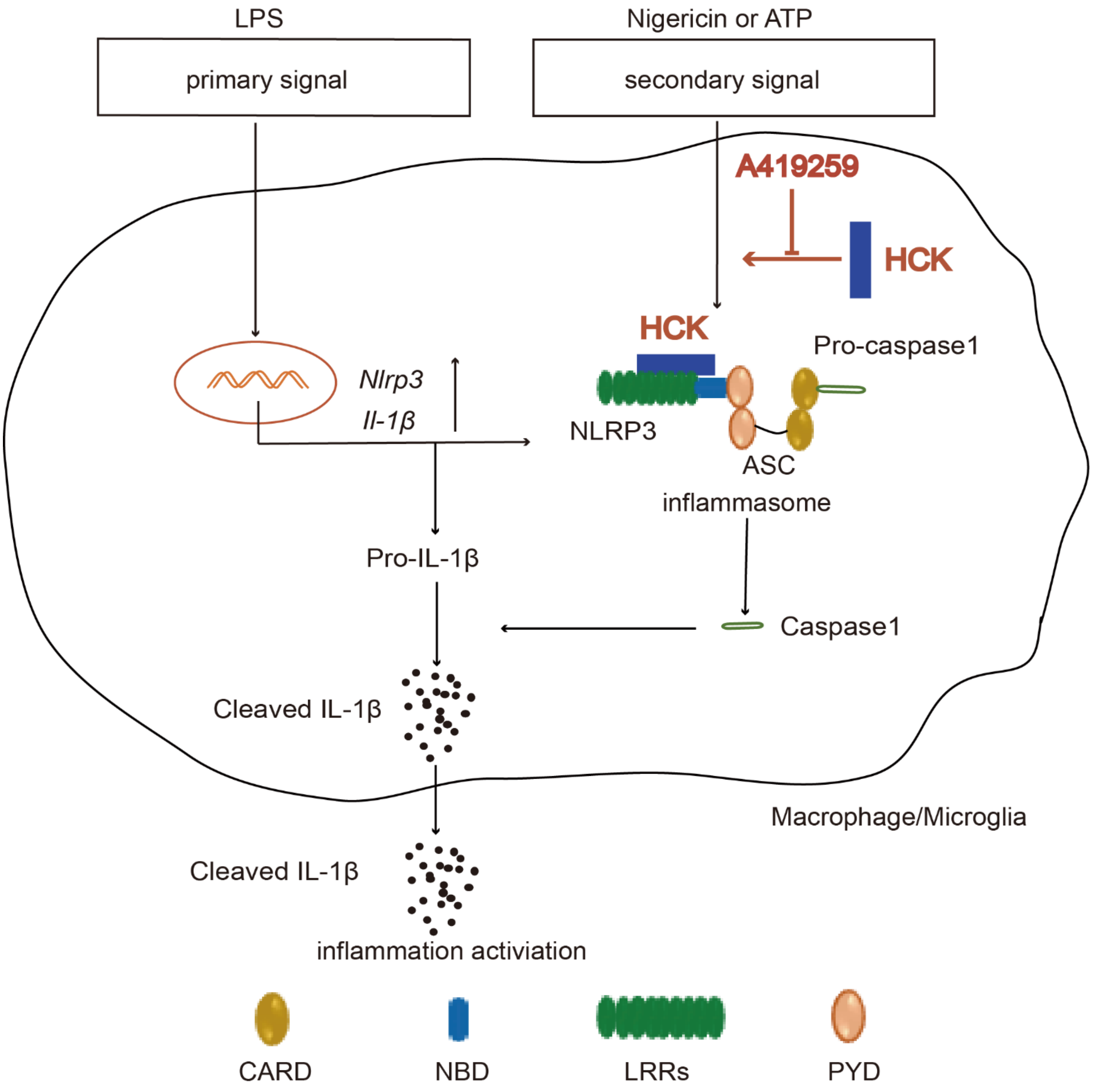

Supplementary Figure 5. Activation of HCK results in NLRP3 inflammasome complex assembly and activation. HCK was phosphorylated and bound to NLRP3 when the macrophages were stimulated by LPS. In the presence of the secondary signal, HCK promoted NLRP3 inflammasome complex assembly and activation, and subsequently, Pro-IL-1β was cleaved by Cleaved caspase1 and was released from the cells. A419259 inhibited HCK phosphorylation and blocked the assembly of the NLRP3 inflammasome complex.
